# Evaluating Early Precursors of Academic Skills: Preliminary Validation of a Touchscreen-Based Digital Assessment in Preschoolers

**DOI:** 10.3390/jintelligence14010004

**Published:** 2026-01-01

**Authors:** Davide Apicerni, Paolo Stievano, James Dawe, Sergio Melogno, Lina Pezzuti

**Affiliations:** 1 Department of Dynamic and Clinical Psychology and Health Studies, Sapienza University of Rome, 00185 Rome, Italy; davide.apicerni@uniroma1.it (D.A.); paolo.stievano@uniroma1.it (P.S.); 2 Local Health Authority Rome 2 (ASL ROMA 2), 00182 Rome, Italy; 3 Department of Humanities and Social Sciences, Universitas Mercatorum Telematic University, 00186 Rome, Italy; james.dawe@unimercatorum.it; 4 Faculty of Psychology, “Niccolò Cusano” University of Rome, 00166 Rome, Italy; sergio.melogno@unicusano.it

**Keywords:** preschool children, school readiness, early literacy, early numeracy, digital assessment, tablet-based tasks, psychometric validation

## Abstract

Early identification of cognitive precursors to literacy and numeracy is essential for promoting school readiness and preventing later learning difficulties. Digital assessment tools using touchscreen technology offer advantages of engagement, standardization, and efficiency. This study reports preliminary findings on the Digital Assessment for Preschoolers—Tool (DAP-T), a touchscreen-based battery for preschool children. A sample of 105 children (M = 61.43 months, SD = 10.38; age range = 38–72) completed eight tasks assessing visuomotor integration, literacy (letter knowledge, phonological awareness, notational awareness, Rapid Automatized Naming), and numeracy (non-symbolic quantity comparison, quantity recognition, counting, cardinality). A subsample (*n* = 47–61, depending on the measure) also completed the paper-based criterion tasks used for concurrent validity analyses. Item difficulty and discrimination, internal consistency (McDonald’s ω), concurrent validity, and factorial structure (SEM) were assessed. Results showed medium-to-low difficulty, age-related performance increases, and good discrimination in most tasks. Reliability was high (ω = 0.713–0.966), and correlations with criterion measures ranged from ρ = 0.52 to 0.95. The DAP-T showed promising psychometric properties as a rapid, standardized tool to detect early difficulties and guide targeted interventions.

## 1. Introduction

Learning is a process that begins at the very start of an individual’s life and develops over the years through multiple pathways, shaped by the types of experiences encountered within the contexts in which the individual is embedded (Bronfenbrenner & Morris, 2007). School represents an environment rich in experiential opportunities that enable children to learn in a variety of ways. Even during the preschool period, children attending early childhood education settings face numerous developmental challenges that allow them to acquire foundational skills and competencies. These are fostered through play-based activities as well as through vertical relationships, with teachers and caregivers, and horizontal relationships, with same-age peers (Vygotsky & Cole, 1978). Research highlights how play-based learning supports cognitive, emotional, and social development by allowing children to explore, interact, and construct meaning within shared contexts (Hedges & Cooper, 2018). Peer relationships formed during play not only enhance socialization and collaboration but also contribute to the acquisition of academic skills across developmental domains (Russo, 2009). Moreover, intentional and relational teacher involvement in play can enrich children’s conceptual understanding, helping bridge everyday experiences with more abstract forms of knowledge (Kausar et al., 2024). Collectively, these early play experiences support key developmental processes—particularly in language, cognition, and emotional regulation—that provide the foundation for later academic abilities such as reading, writing, and mathematics in the primary years.

The extent to which a child has developed a set of basic competencies that serve as resources for success in primary school is defined by the concept of school readiness (Beecher et al., 2018), which is conceptualized as a multidimensional construct encompassing foundational academic competencies (particularly early mathematics and reading skills), attentional and self-regulatory abilities, and socio-emotional skills that children possess upon entering primary school. Longitudinal evidence indicates that early academic and attentional skills are among the strongest predictors of later academic achievement, whereas socio-emotional competencies, although important for classroom adaptation, exert a less direct influence on academic performance (Duncan et al., 2007). Children may display varying levels of school readiness, with differences emerging in emotional and behavioral regulation skills as well as in the cognitive abilities that underpin the development of academic competencies (Magnuson et al., 2004; Majzub & Rashid, 2012). Given that school readiness in preschool is a strong predictor of subsequent academic performance, identifying potential indicators of difficulty at this stage is essential to provide timely support and foster successful educational trajectories.

### 1.1. Precursors of School Learning in Preschool Age: Domain-General and Domain-Specific Skills

During the preschool years, certain cognitive abilities serve as fundamental precursors to school learning. Their development at this stage constitutes a predictive indicator of the future instrumental skills that will be acquired during the primary school years. Some of these abilities play a domain-general role, as they are cognitive skills involved across both literacy (reading and writing skills) and numeracy. These include attention and executive functions (Miyake et al., 2000; Diamond, 2013), intelligence level, expressive and receptive language, motor skills, visuomotor integration skills, rapid naming abilities, visuospatial skills, and working memory. Among the domain-specific abilities related to learning, grapheme–phoneme association, morphological awareness, rapid naming, and notational awareness are linked to reading and writing skills; whereas quantity comprehension, recognition of written numbers, and understanding of the concept of number linearity are among the specific precursors of the mathematical domain (Koponen et al., 2013; Passolunghi & Lanfranchi, 2012; Hjetland et al., 2017; Peng & Fuchs, 2016; Usai et al., 2018; ISS, 2022).

#### 1.1.1. The Development of Literacy Skills: Precursors to Reading and Writing

In the preschool years, the precursors of reading and writing skills are closely interconnected, particularly during the initial stages of literacy. The concept of early literacy refers to the idea that learning to read and write is a progressive process that begins in early childhood (Whitehurst & Lonigan, 1998). Several theoretical models have described this developmental trajectory. According to Frith’s (1986) stage model, reading development unfolds through three sequential phases. In the first, the logographic stage, typical of early childhood, children recognize words primarily based on global visual cues, without analyzing their phonological or orthographic structure. In the second, the alphabetic stage, children shift to phonological decoding, acquiring phonological awareness—that is, the ability to reflect on grapheme–phoneme correspondences—and developing the capacity to segment words into phonemic units, which enables them to read unfamiliar words using letter–sound conversion rules. Finally, in the orthographic stage, readers are able to recognize words automatically and rapidly thanks to a consolidated orthographic memory, allowing them to process both the regularities and exceptions of the writing system.

An alternative framework is the Simple View of Reading (Gough & Tunmer, 1986), which posits that reading ability is the product of word recognition (decoding) and language comprehension. This model has since been expanded to highlight how broader cognitive factors (e.g., executive functions, verbal fluency, and vocabulary) and socio-emotional–behavioral factors (e.g., motivation and self-regulation strategies) also influence the development of reading skills (Duke & Cartwright, 2021). Furthermore, the dual-route model (Coltheart, 2006) suggests that reading operates through two distinct pathways: a phonological route, based on grapheme–phoneme conversion (useful for novel or unfamiliar words), and a lexical route, which allows for the direct recognition or production of known words through access to orthographic and phonological representations in long-term memory. In the early stages of learning, the phonological route is predominantly activated; as children become more proficient readers, both routes begin to operate in parallel, jointly supporting the efficiency and automatization of decoding processes.

Compared to reading, the development of writing skills has been the focus of fewer empirical investigations; nonetheless, several theoretical models have outlined its progression. The classic model proposed by Ferreiro and Teberosky (1982) describes writing competence as the outcome of a developmental process progressing through qualitatively distinct stages—from pre-syllabic, to syllabic, to alphabetic, and finally to orthographic—during which children gradually construct awareness of the relationships between spoken and written language. More recent perspectives, however, have proposed a multidimensional view of emergent writing. For example, Puranik and Lonigan (2014) introduced a three-factor model distinguishing between conceptual knowledge (functions and conventions of writing), procedural knowledge (basic graphomotor and orthographic skills), and generative knowledge (text production beyond the single word). Similarly, Bigozzi et al. (2016) proposed a multidimensional framework incorporating phonological awareness, conceptual knowledge of the writing system, and textual competence, with particular emphasis on the early mastery of orthographic conventions and writing rules. These contemporary approaches underscore that writing development involves a wide range of skills—not only phonological, but also conceptual and functional—emerging in the early years of life, with important implications for the early identification of children at risk for learning difficulties.

Taken together, these theoretical models highlight that both reading and writing rely on several foundational abilities, such as recognizing letter–sound correspondences and consciously distinguishing and reflecting on the sounds within words (phonological awareness). Reading development is further supported by skills such as phonemic segmentation, the construction of a stable orthographic memory, and language comprehension, as well as broader cognitive functions including vocabulary, executive functions, verbal fluency, and self-regulation strategies (Frith, 1986; Gough & Tunmer, 1986; Coltheart, 2006; Duke & Cartwright, 2021). For writing, in addition to phonological awareness, key contributors include graphomotor and orthographic abilities, conceptual knowledge of writing conventions, textual competence, and the early mastery of orthographic rules (Ferreiro & Teberosky, 1982; Puranik & Lonigan, 2014; Pinto et al., 2012; Bigozzi et al., 2016; Neri & Pellegrini, 2017). In this sense, developmental models underscore that literacy acquisition requires the integration of phonological, orthographic, conceptual, and functional skills from the earliest years, with critical implications for the early identification of children at risk for learning difficulties.

A substantial body of empirical research has further investigated the cognitive abilities that, in the preschool years, predict the development of literacy. Among these, phonological awareness, grapheme–phoneme association, short-term memory, notational awareness (i.e., the ability to process forms of writing resembling conventional orthography in relation to the phonemic strings composing words), vocabulary, visual–verbal association learning, morphological awareness, and Rapid Automatized Naming (RAN) have been identified as the skills most strongly associated with decoding development (Kirby et al., 2010; Brown, 2014; Bigozzi et al., 2016; van Viersen et al., 2017; Psyridou et al., 2021; Protopapas, 2019; Ecalle et al., 2023). These same abilities—particularly phonological awareness, RAN, visual–verbal associations, notational awareness, and short-term memory—have also been found to predict orthographic competence (Catts et al., 2015; Melby-Lervåg et al., 2012; Maniscalco et al., 2015; Mercugliano et al., 2025). The key role of these variables in literacy development has been confirmed by longitudinal studies across different languages (Landerl et al., 2019; Wealer et al., 2022; Landerl et al., 2022). Regarding reading, RAN and notational awareness have consistently emerged as particularly strong predictors (Norton & Wolf, 2012; T. G. Scalisi et al., 2013), with combined deficits in these two areas (the so-called “double deficit”) characterizing the most at-risk reading profiles (T. G. Scalisi et al., 2005; Kirby et al., 2010; Furnes et al., 2019). Finally, notational competence has also been shown to be a robust predictor of writing skills (Pinto et al., 2017).

#### 1.1.2. The Development of Numeracy Skills: Symbolic and Non-Symbolic Precursors of Number

One of the most influential theoretical frameworks in the study of the development of mathematical abilities from early childhood is the Triple Code Model (Dehaene, 1992; Dehaene & Cohen, 1996; Skagenholt et al., 2021). This neurocognitive model identifies three distinct systems of numerical representation—the visual symbolic code (Arabic numerals), the verbal symbolic code (number words), and the non-symbolic code for the approximate estimation of numerosity—each associated with specific neural networks and cognitive processes dedicated to numerical cognition.

More recently, the Triple Code Model has been complemented by an innovative perspective suggesting that the development of mathematical abilities may be guided by a form of language of thought (LoT) underlying both arithmetic and geometric acquisitions. According to this view, basic cognitive operations—such as repetition, concatenation, and recursion—enable the generation and manipulation of numerical and geometric structures from early childhood (Dehaene et al., 2025). Consequently, the precursors of numeracy skills include a range of cognitive abilities, such as procedural, conceptual, and symbolic competencies related to counting, executive functions, and visuospatial memory capacities. Certain domain-general abilities, shared with the literacy domain, also contribute to the acquisition of mathematical competencies; these include vocabulary, sustained attention (Cahoon et al., 2021), nonverbal intellectual abilities, and phonological awareness (Nogues & Dorneles, 2021).

The acquisition of numerical skills involves both symbolic and non-symbolic competencies (Raghubar & Barnes, 2017). Symbolic skills include understanding the meaning of numbers, numerical sequencing, knowledge of Arabic numerals, the cardinal principle (the understanding that the last number counted represents the total number of objects in a set), the ability to associate numerical symbols with quantities, number identification and naming, quantity discrimination, and counting. Non-symbolic skills, on the other hand, encompass the ability to manipulate and transform quantities, compare magnitudes, sense numbers, and discriminate between different quantities (Howell & Kemp, 2010; Tobia et al., 2016). Among the latter, in recent years, particular attention has been devoted to the Approximate Number System (ANS); that is, the ability to compare two sets of elements (e.g., black dots) with different quantities and quickly determine which is larger without resorting to counting. This mechanism, considered innate and non-symbolic, appears to play a predictive role in the development of arithmetic skills in preschool children (Mazzocco et al., 2011; Vallortigara & Panciera, 2014; Raghubar & Barnes, 2017; Bernabini et al., 2020).

### 1.2. Assessment Tools for Evaluating the Precursors of Literacy and Numeracy Skills

Given the complexity of the processes that collectively underpin the development of instrumental skills related to academic learning, the assessment of literacy and numeracy precursors in preschool age requires a multilevel approach capable of providing a comprehensive profile of developmental trajectories. While some competencies are domain-general and span across various areas of learning, others are domain-specific cognitive skills (Cirino et al., 2016; Purpura et al., 2017).

In the international context, numerous validated psychometric instruments are available for the assessment of cognitive domains and learning precursors in preschool-aged children. Among the most widely used tools for evaluating domain-general abilities are the Griffiths III Scales of Child Development (Green et al., 2020; Taddei et al., 2023; Pino et al., 2024), the Wechsler Preschool and Primary Scale of Intelligence—Fourth Edition (WPPSI-IV) (Wechsler, 2012; Saggino et al., 2019), and the Leiter International Performance Scale—Third Edition (Leiter-3) (Roid et al., 2013) for the assessment of general cognitive abilities. The NEPSY-II battery is also widely used for the evaluation of executive functions, language, memory, social perception, and visuospatial skills (Davis & Matthews, 2010; Cardillo & Mammarella, 2015; Rosenqvist et al., 2017), while the Developmental Test of Visual–Motor Integration (VMI) assesses visuomotor integration (Dibek, 2012; Bonichini, 2017). Commonly used tools for language assessment include the Peabody Picture Vocabulary Test (PPVT) (Eigsti, 2021), the Clinical Evaluation of Language Fundamentals—Fourth Edition (CELF-4) (Semel et al., 2003; Paslawski, 2005), and the Italian BVL 4-12 battery for linguistic profiling (Marini et al., 2015; Guarini et al., 2016). 

With regard to literacy precursors, instruments such as the Test of Preschool Early Literacy (TOPEL) (Lonigan et al., 2007), the Italian SPEED battery (Savelli et al., 2013), the PAC-SI (T. Scalisi et al., 2009), the Comprehensive Test of Phonological Processing—Second Edition (CTOPP-2) (Dickens et al., 2015), and the Compiti di Metafonologia (CMF) (Marotta et al., 2008) allow for the evaluation of emergent literacy skills, such as phonological awareness, alphabet knowledge, and RAN, with an in-depth assessment of phonological abilities.

For assessing early mathematical skills, relevant tools include the Italian BIN 4-6 battery for numerical intelligence (Molin et al., 2006), the SNUP test (Tobia et al., 2017), and the ENT-R (Menacho et al., 2024).

As an alternative to traditional paper-and-pencil instruments, recent years have witnessed the development of numerous tests designed to assess cognitive precursors of learning through touchscreen devices such as tablets and laptops. These technologies, which are associated with promising results, provide opportunities to evaluate neurocognitive developmental skills via software that delivers a game-like experience familiar to children (Bhavnani et al., 2021; Tenorio Delgado et al., 2016; Bastianello et al., 2023; Mukherjee et al., 2024; Park et al., 2021; Carson et al., 2011; Clemens et al., 2019; Gaggi et al., 2012).

With respect to the paper-and-pencil traditional approach, the advantages of touchscreen-based assessment are manifold. These approaches may enhance children’s compliance during administration, as their playful nature proves both engaging and motivating. Moreover, interaction with the electronic device inherently involves the child’s fine motor skills as well as executive functions (Dan & Pelc, 2019). Additional benefits of digitized assessment over traditional methods include the provision of immediate and individualized feedback—unlike paper-based procedures, which require greater time and economic resources—and the precision and efficiency afforded by standardized procedures (Tonelli et al., 2018). Finally, from a research perspective, digital technologies offer advantages in terms of data collection and storage, which can be carried out more efficiently than with paper materials (Bridgeman, 2009), including the possibility of remote administration (Lampis et al., 2024).

However, despite these advantages, most existing digital tools have been designed to target specific skills in isolation, often focusing exclusively on either literacy or numeracy. This underscores the need for comprehensive instruments capable of jointly assessing multiple cognitive domains relevant to school readiness in a standardized and engaging digital format.

### 1.3. The Present Study

The present study introduces the Digital Assessment for Preschoolers—Tool (DAP-T), a battery of digitized tasks developed for administration via touchscreen tablet devices. The DAP-T was designed to provide an integrated assessment of early cognitive precursors of learning across domains. Specifically, it evaluates literacy skills (phonological awareness, alphabet knowledge, rapid automatized naming (RAN), and notational awareness), numeracy skills (non-symbolic abilities such as the Approximate Number System (ANS) and quantity recognition, as well as symbolic abilities such as cardinal skills), and visuomotor integration abilities.

The DAP-T is aweb-based software accessible via a web platform and is compatible with multiple operating systems (e.g., iOS, Android, Windows, MacOS). The application presents all stimuli, records touch responses and vocal answers, and stores raw data in a format suitable for subsequent statistical analysis. The software was developed by an external provider under the supervision of the research team; detailed implementation features (e.g., specific programming language) are proprietary and not directly relevant to the psychometric aims of the present study. At the time of writing, the DAP-T is available in Italian for research purposes only. Researchers interested in using the instrument in collaborative projects may contact the first author for further information about access conditions.

To examine the preliminary psychometric properties of the DAP-T, several analytic steps were undertaken. First, an item analysis was conducted to evaluate item difficulty and discrimination indices, following the principles of classical test theory. We hypothesized that most items would fall within the acceptable range of difficulty (30–70% correct responses) and yield discrimination indices at or above the recommended threshold of 0.40, indicating adequate item functioning (Barbaranelli & Natali, 2005).

Second, criterion validity was tested by comparing DAP-T tasks with standardized external measures already validated in the literature (John & Benet-Martínez, 2000). We expected positive and significant correlations between literacy tasks (phonological awareness, alphabet knowledge, RAN, and notational awareness) and established literacy measures, between numeracy tasks (non-symbolic and symbolic abilities) and measures of early numerical competence, and between the visuomotor task and a standardized visuomotor measure.

Third, the internal structure of the battery was investigated through confirmatory factor analysis (CFA; Floyd & Widaman, 1995; Gallucci & Leone, 2012), and internal reliability was estimated using McDonald’s omega coefficients (Dunn et al., 2014). We hypothesized that the CFA would support the presence of a general latent factor underlying both literacy and numeracy tasks, reflecting a core cognitive ability, and that all subscales would demonstrate adequate internal consistency (ω ≥ 0.70; Tavakol & Dennick, 2011; Dunn et al., 2014).

## 2. Materials and Methods

The study was conducted in accordance with the Declaration of Helsinki and approved by the Institutional Review Board (or Ethics Committee) of Sapienza University of Rome (protocol code 249/2024; date of approval 12 December 2024). Participant recruitment was conducted in three educational institutions located in central Italy that had previously established agreements with the University Department. The process began with two preliminary meetings—one with the teaching staff and another with parents and/or legal guardians—both aimed at presenting the research project and the study’s aims. Written informed consent was obtained from parents/legal guardians for every participating child; participation could be withdrawn at any time without penalty and no identifying information was included in the analytic dataset. The informed consent form also included a brief sociodemographic section designed to collect information about the language predominantly spoken at home and the presence of any previous neurodevelopmental diagnoses. All children whose families provided consent were included in the administration phase, and no child was excluded prior to data collection. Since the aim of the study was to explore the psychometric properties of the battery within the framework of a validation project, participants who were not native Italian speakers, or who had a diagnosis of developmental disorders, neurological conditions, or other pre-existing clinical conditions that could compromise the regular execution of the tasks, were excluded from the data analyses. Additionally, children older than 72 months were excluded to ensure greater sample homogeneity with respect to preschool age.

The study involved 126 Italian children attending three preschools, aged between 38 and 76 months (M = 61.66, SD = 10.55), and enrolled in the first, second, or third year of preschool. Based on the exclusion criteria, one participant was excluded due to a previous neurological diagnosis, and twenty participants were excluded based on the age criterion (>72 months). The final analytic sample consisted of 105 children (M = 61.50 months, SD = 10.36; IQR = 54–69 months), including 56 girls (53.3%). The demographic characteristics of the sample are reported in Table 1.

The administration activities were conducted in a dedicated school room, in a one-to-one setting. The administration sessions were conducted by the first author of the present work (co-author of the DAP-T instrument) and by trained psychology students. Verbal assent was also obtained from all participating children, with the request adapted to each child’s verbal ability, prior to the start of the administration activities, in order to avoid stressful conditions and to promote a climate of voluntary participation. Each participating child completed one administration session of the DAP-T tasks on touchscreen tablets with screen sizes ranging from 10.5 to 11 inches, with the session lasting about 30 min on average.

Furthermore, of the 105 children included in the analytic sample, 65 completed the paper-based criterion measures. This number was determined by practical constraints. Because the time made available by the schools for additional assessment sessions was limited and only a portion of the research team was qualified to administer the paper-based measures, it was possible to test 65 children during the study period. All eligible and available children were included, and no further selection criteria were applied.

The administered tasks were as follows:

*Literacy domain*
  - Letter Game: This task consists of 16 items and assesses letter knowledge. It involves the on-screen presentation of letters and syllables, which the child is required to recognize and name. The software records the child’s vocal responses, including response times. Accuracy scores are assigned by the examiner during the task or, when necessary, by reviewing the audio recording afterward.
    For letters, accuracy scoring ranges from 0 to 2: 0 for no response or incorrect response, 1 if the letter is pronounced as the initial of a word (e.g., “A” → “ape” [bee]), and 2 if the letter is correctly named.
    For syllables, scoring ranges from 1 to 3: 1 if letters are read separately (e.g., “MA” → “m”—“a”), 2 if the syllable is pronounced as the initial of a word (e.g., “MA” → “mamma” [mom]), and 3 if it is read correctly as a whole syllable (e.g., “MA” → “ma”). For the purposes of the present analyses, we considered only accuracy scores. Although the software also records response times, temporal parameters were not included in the analyses because the validation focused primarily on accuracy-based performance indicators.
  - Rhyme Game: This task, designed to assess phonological awareness, consists of 16 items and is structured in three modalities: in the first two, the child selects—among multiple options—the word that rhymes with a target word; in the third, the child identifies which of the options does not rhyme with the others. Before each item, the child is asked to name the three pictures displayed on the screen to verify the correspondence between the visual stimulus and its lexical label. When necessary, the examiner provides the correct label to ensure that the child is using the intended word forms, which is essential for determining rhyme-based associations. In the first modality, the child chooses and touches the picture whose name rhymes with the target word. In the second modality, the child selects the two pictures whose names rhyme with each other. In the third modality, the child identifies and touches the picture whose name does not rhyme with the other two. Responses are provided via touchscreen, and an accuracy score is calculated (0 for incorrect responses and 1 for correct responses).
  - Rapid Automatized Naming (RAN) of Pictures: This task assesses rapid naming ability. The child is required to name, as quickly and accurately as possible, a matrix of 80 images depicting common objects with high-frequency bisyllabic names (Rinaldi et al., 2003). Both execution time and an audio recording of the performance are collected. One point is awarded for each correctly named item (zero points for incorrect naming). For the purposes of performance evaluation, the primary parameter considered is the number of correct responses produced within 60 s. This time frame was chosen because the average completion time in our sample was approximately 108 s; therefore, the first 60 s constitute a meaningful and comparable window to index naming speed. In addition, a 60 s criterion is consistent with the Rapid Naming Task of the RIAS-2 (Reynolds Intellectual Assessment Scales, Second Edition; Pezzuti et al., 2021), which served as the criterion measure for the RAN task.
  - Drawing and Writing Game: This task assesses notational awareness, namely children’s ability to understand the correspondence between the phonemic string characteristics of words and their written representation. The task consists of 15 items and includes five phases in which the child is asked to draw specific stimuli and write their names. The task can be carried out either with a digital pen, e.g., Apple Pencil (Apple Inc., Cupertino, CA, USA) compatible with the touchscreen device or with a whiteboard marker on printed and laminated sheets. Before each item, the examiner asks the child to name the picture to ensure knowledge of the lexical label and provides the correct label when needed. Children complete the items using the pen-based modality assigned for that session.
  - Phase 1 (three items). The child is shown a written word in uppercase print and an illustration. The child is asked to discriminate between the two by touching the written word. One point is awarded for a correct response; zero points are assigned for omissions or for selecting the illustration.
  - Phase 2 (three items). The child is asked to draw a target stimulus (e.g., a “person”) and to write its name. Scoring follows a 0–2 scale: 2 points if both the drawing and the written label are produced and the written string contains at least three letter-like symbols (reversed letters are accepted); 1 point if both drawing and writing are present but the written production contains only one or two letter-like symbols; 0 points if the child produces only the drawing or if the written production contains no recognizable letter-like symbols.
  - Phase 3 (three items). Each item presents a pair of pictures with lexical labels differing in length (e.g., Frigorifero [refrigerator] vs. Ago [needle]). The child is asked to write the name of each image. Scoring is as follows: 2 points if both written strings contain at least three letter-like symbols and the string for the long word is meaningfully longer than that for the short word (e.g., at least a 2:1 ratio); 1 point if both strings contain at least three letter-like symbols but do not differ substantially in length; 0 points if no writing is produced or if either string contains fewer than three letter-like symbols.
  - Phase 4 (three items) and Phase 5 (three items). These phases involve pairs of stimuli whose lexical labels differ only in the final letter—masculine vs. feminine (e.g., Nonno [grandfather] vs. Nonna [grandmother]) and singular vs. plural (e.g., Orso [bear] vs. Orsi [bears]). The child is asked to write the name of each picture. Scoring is based on whether the child differentiates the written forms only in the final segment: 2 points if both written strings contain at least three letter-like symbols and differ exclusively in the final symbols; 1 point if both strings contain at least three letter-like symbols but differ in additional segments (or are identical); 0 points if no writing is produced or if either written string contains fewer than three letter-like symbols.
*Numeracy domain*
  - Golden Tokens Game—Quantity Recognition: This task, consisting of eight items, assesses non-symbolic numerical cognition. The child watches an animation in which a certain number of tokens fall into a piggy bank and is then required to replicate the same quantity in a virtual piggy bank by dragging tokens with their finger on the touchscreen. The software records the response and provides an accuracy score (0–1 points) as an indicator of non-symbolic numerosity cognition.
  - Quantity Comparison (Approximate Number System—ANS) Task: This task, consisting of ten items, involves the simultaneous and tachistoscopic presentation (2000 ms) of two boxes containing different numbers of black dots. The numerosities displayed in the two boxes follow Weber’s law, whereby comparing two quantities is easier when their ratio is larger (e.g., 10:1) and becomes more difficult as the ratio decreases (e.g., 10:8) (Feigenson et al., 2004).
  - The child is required to indicate, by touching the touchscreen, the box containing more dots (first phase) or fewer dots (second phase). The software records the responses and computes both an accuracy score (0–1 points) and a response time. This task was designed to provide a measure of the approximate number system.
  - Ball Jar Game—Cardinal Skills: Based on the caterpillar task (Hannula & Lehtinen, 2005), this task consists of twelve items in which the child hears a number and is required to drag the corresponding number of balls into a virtual jar using their finger on the touchscreen. The software records both an accuracy score (maximum 58 points) and the highest cardinal value reached (maximum 10) as indicators of counting and cardinality skills. For the purposes of this study, only the accuracy parameter was considered.
*Visual–Motor integration*
  - Pathways Game: This task, consisting of six items, presents paths to be traced either with a digital pen on the touchscreen or with a whiteboard marker on printed and laminated sheets. The goal is to follow each path, of varying difficulty, from start to finish as quickly as possible, aiming to touch the target elements along the way while avoiding crossing the boundaries or lifting the pen tip.
    An accuracy parameter is recorded, defined as the total number of target elements touched (maximum 104), as well as execution time and errors, which are counted as the number of pen lifts from the surface and deviations from the path. For the purposes of data analysis, only the accuracy parameter was considered.

The subsample involved in the administration of the criterion measures completed a battery of previously validated tests, selected as gold-standard measures, in order to assess concurrent validity. Although 65 children were originally assigned to complete these tasks, the final sample sizes vary across tasks (*n* = 47–61). This variability reflects the challenges of testing preschool children, as some participants were unable to complete all tasks due to fatigue or loss of attention, while in other cases time constraints within the school setting limited the administration of specific tasks. The size of the subsamples completing the criterion tasks ranged from 47 to 61 participants.

The paper-based criterion measures included:

- Tasks 1 “Letter Recognition,” 2 “Letter Naming,” and 3 “Letter Writing” (SPEED—Screening Prescolare Età Evolutiva—DISLESSIA; Savelli et al., 2013). These tasks assess the ability to recognize, name, and write the letters of the alphabet. Uppercase printed letters are presented one at a time, and the child is asked to identify the presented letter, say its name, and write it on a sheet of paper. An accuracy score (0–1) is assigned for each item. The number of participants completing these tasks was 59, 55, and 54, respectively.
- Rhyme Recognition Task (CMF; Marotta et al., 2008). This task, which measures phonological awareness, requires children to select, from the options provided, the image whose name rhymes with that of a target picture. An accuracy score (1 point for each correct response) is assigned. The subsample completing this task, after outlier removal, consisted of 53 participants.
- Rapid Naming Task (RIAS-2—Reynolds Intellectual Assessment Scales, Second Edition; Pezzuti et al., 2021). This task, which measures rapid automatized naming (RAN), presents a grid containing images of animals and objects familiar to children. The child is asked to name them as quickly as possible, proceeding in order from left to right. One accuracy point is assigned for each correctly named item. For the purposes of data analysis, the final score considered was the total number of correct responses within 60 s. The subsample completing this task, after outlier removal, consisted of 47 participants.
- Quantity Comparison Task (BIN 4-6—Battery for the Assessment of Numerical Intelligence in Children Aged 4 to 6 Years; Molin et al., 2006). This task measures the ability to discriminate between two groups of elements with different numerosities. Children are presented with cards showing groups of black dots contained within two rectangles and are asked to compare the quantities in each group, indicating the one containing the larger number. One point is awarded for each correct response, and the parameter of interest is the total accuracy score. The subsample completing this task, after outlier removal, consisted of 56 participants.
- Graphic Elements Counting Task (BVN 5-11—Neuropsychological Assessment Battery for Developmental Age; Bisiacchi et al., 2005). This task measures counting ability and the capacity to determine the numerosity of a group of elements. Children are presented with cards containing groups of dots and are asked to count them and state the total number of elements. One point is awarded for each correct response, and execution time for each item is recorded. For the purposes of statistical analyses, the parameter considered was the total accuracy score. The subsample completing this task, after outlier removal, consisted of 53 participants.
- Short Form of the Developmental Test of Visual–Motor Integration (VMI; Beery, 2015). This task measures visuomotor integration skills. Children are asked to copy a series of geometric shapes that become progressively more complex. One point is awarded for each correct reproduction. The parameter of interest is the total accuracy score. The subsample completing this task, after outlier removal, consisted of 61 participants.

### Data Analysis

Preliminary descriptive analyses were conducted to examine the characteristics of the score distributions. The results, presented in Table 2, show that the distribution of scores for most variables did not follow a normal pattern (Shapiro–Wilk test statistically significant); consequently, non-parametric statistical tests were employed.

To investigate the effect of gender, the non-parametric Mann–Whitney U test was used. To explore the effect of age on the abilities assessed by the DAP-T tasks, we conducted linear regression analyses using age in months (38–72 months) as the predictor. Regression models allowed us to estimate the magnitude of age effects and to quantify the proportion of variance explained by age in each task.

To examine the characteristics of the items, item difficulty and the discrimination index (DI) were calculated for DAP-T tasks presenting both correct and incorrect responses. Item difficulty was assessed by calculating the percentage of correct responses both for the overall sample and for each age group, considering percentages between 30% and 70% as acceptable, percentages above 70% as indicative of very easy items, and percentages below 30% as indicative of difficult items (Barbaranelli & Natali, 2005; D’Sa & Visbal-Dionaldo, 2017).

The DI for each item was calculated as the difference between the proportion of correct responses in the top 27% and the bottom 27% of participants. The use of the 27% cut-off follows Kelley’s (1939) recommendation for maximizing group differences. Items with DI ≥ 0.40 were considered to have good discrimination; values between 0.30 and 0.39 indicated items functioning well but possibly requiring minimal revision; values between 0.20 and 0.29 indicated sufficient discrimination; and DI ≤ 0.19 suggested the need for substantial revision or elimination (Crocker & Algina, 1986).

For the bivariate correlation analyses between DAP-T scores and the criterion measures used to examine concurrent validity, Spearman’s rho coefficients were calculated and corrected for the effect of attenuation (Cohen et al., 2013; Barbaranelli & Natali, 2005). Effect sizes were interpreted according to Cohen’s (2013) criteria: small (r = 0.10), medium (r = 0.30), and large (r = 0.50).

For the analysis of internal consistency, McDonald’s omega coefficients were computed (Dunn et al., 2014). In line with the recommendations of Dunn et al. (2014), this study used McDonald’s ω coefficient instead of Cronbach’s α, as it provides a more accurate estimate of reliability in the absence of tau-equivalence, more faithfully reflecting the variance attributable to the latent construct.

Finally, to investigate the factorial structure of the battery, confirmatory factor analyses (CFA) were conducted within a structural equation modeling (SEM) framework using the Robust Maximum Likelihood (MLR) estimation method (Kline, 2023). Although SEM typically benefits from larger samples, its application was considered appropriate here given the model’s parsimony, the theoretical grounding of the constructs, and simulation evidence showing that CFA can yield stable estimates with samples around 100 when factor loadings are moderate to high and the model is well specified (Shevlin & Miles, 1998).

Descriptive analyses, analysis of variance (ANOVA), bivariate correlations, and SEM were performed using the Jamovi statistical software (version 2.6; The Jamovi Project, 2024, Sydney, Australia) with additional modules (Fox & Weisberg, 2023; Revelle, 2023; Gallucci & Jentschke, 2021; Rosseel, 2019; Epskamp et al., 2019). Item difficulty analyses and the calculation of discrimination indices (DI) were conducted using Microsoft Excel (Microsoft Corporation, 2024, Redmond, WA, USA).

## 3. Results

### 3.1. Item Analysis

Item difficulty and discrimination analyses were conducted for the Letter Game, Rhyme Game, Drawing and Writing Game, Quantity Comparison Task (ANS), Golden Tokens Game, and Ball Jar Game, as these tasks primarily consist of items evaluated in dichotomous terms of pass/fail. In some of these tasks, item scores include multiple levels; however, they still reflect a dichotomous condition of success or failure. Conversely, for the RAN and visuomotor integration tasks, overall accuracy derives from the integration of both correctness and execution time.

The results from the item difficulty analysis and the calculation of the discrimination index (DI) (available in Appendix A) indicated that the item analysis revealed a consistent age-related trend across all tasks, with higher success rates in older children and progressively lower difficulty with age. For the Letter Game, the overall mean success rate was 68% (SD = 7.1; range = 58.3–87.4%), ranging from 21.7% in the 38–48-months group to 83.3% in the 61–72 months group; discrimination indices (DIs) were generally good (0.48–1.00), with only one critical item in the two older groups. The Rhyme Game showed moderate difficulty in the total sample (M = 60.6%, SD = 14.1), with mean success rates of 34.4%, 49%, and 70.3% across the three age groups; DI values were satisfactory (0.26–0.96), though five items were poorly discriminating in the youngest group and one in the intermediate group. The Drawing and Writing Game had high overall success (M = 85.3%, SD = 5.9), with rates increasing from 38.3% in the youngest group to 99% in the oldest; while DIs were good in the younger groups, a ceiling effect was evident in the 61–72 months group, where all items had DI < 0.20. In the Quantity Comparison (ANS) Task, the mean success rate was 86% (SD = 9.7), ranging from 69.4% to 92% across age groups; most items showed satisfactory discrimination (0.22–0.67), though the easiest items had lower DI values, particularly among older children. The Golden Tokens Game demonstrated a mean success rate of 54.2% (SD = 21.1), with increasing performance from 25.8% to 62.8% across the three age groups; DI values were strong overall (0.71–0.96), with good discrimination in all age subgroups. Finally, in the Ball Jar Game, the overall success rate was 72.5% (SD = 14.1), ranging from 21.1% in the youngest to 90.6% in the oldest group. Discrimination was generally good (0.40–1.00), except for the first item, which was too easy (DI = 0.12), and for a subset of items showing floor effects in the youngest group and reduced discrimination in the initial items for the oldest.

### 3.2. The Factorial Structure of the Battery

To examine the latent structure of the tasks assessing precursors of academic learning, two models were tested through confirmatory factor analysis (CFA) conducted within a structural equation modeling (SEM) framework, using the total scores of each task as observed variables. First, a unidimensional model was tested in which all tasks loaded on a single general factor. Subsequently, a nested two-factor model was tested, with two correlated factors representing the literacy and numeracy domains. In this model, the observed variables related to phonological awareness, notational awareness, RAN, and alphabet knowledge loaded on the Literacy factor, while the ANS, quantity recognition, cardinal skills and visuomotor integration variables loaded on the Numeracy factor.

The unidimensional model showed a good fit to the data: AIC = 5130.002; BIC = 5193.697; χ^2^(20) = 23.08, *p* = .285; RMSEA = 0.038; CFI = 0.989; TLI = 0.985; SRMR = 0.038. The two factors model yielded comparable fit indices: AIC = 5132.300; BIC = 5201.303, χ^2^(18) = 22.62, *p* = .255; RMSEA = 0.043; CFI = 0.989; TLI = 0.983; SRMR = 0.038. A direct comparison between the two models revealed no statistically significant differences: ΔRMSEA = −0.005; ΔCFI = 0.000; ΔTLI = 0.002; ΔSRMR = 0.000. Based on the parsimony criterion, the unidimensional model was therefore selected as the simplest and most effective representation of the data structure. Table 3 reports the standardized factor loadings (95% CI), standard errors (SEs), residuals, and *p*-values.

### 3.3. Internal Consistency Analysis of the Tasks

As shown in Table 4, values ranged from 0.713 to 0.966, indicating good-to-excellent reliability for most tasks. In particular, the Letter Knowledge and RAN tasks yielded ω values of 0.964, while the task measuring cardinal skills reached 0.966, reflecting high internal consistency. Values ranging from 0.77 to 0.94 were observed for Golden Tokens (0.772), Rhyme (0.808), Pathways (0.814), and Notational Awareness (0.935), also indicative of good reliability. The only task with a value at the lower bound of the conventional 0.70 threshold was the ANS (0.713), which nevertheless falls within the acceptable reliability range (Gallucci & Leone, 2012).

### 3.4. Concurrent Validity Analysis 

Correlations between the DAP-T tasks and the criterion measures were calculated using Spearman’s ρ coefficient, as score distributions did not meet the normality assumption (significant Shapiro–Wilk test). The observed correlations were also corrected for attenuation due to measurement error. The correction was computed using the classical correction-for-attenuation formula:

$$r_{\mathrm{corrected}}=\frac{r_{\mathrm{observed}}}{\sqrt{\left(rel_{x}\cdot rel_{y}\right)}}$$

where *r*_observed_ represents the observed correlation coefficient, *r*_corrected_ represents the correlation corrected for attenuation, and *rel_x_* and *rel_y_* correspond to the reliability coefficients (Cronbach’s α or McDonald’s ω) of the two measures. Reliability estimates for the DAP-T tasks were computed in the present study using McDonald’s ω, whereas the reliability coefficients for the paper-based criterion measures were obtained from their published test manuals. This procedure provides an estimate of the correlation between the latent true scores, adjusting for unreliability in the observed measures (Barbaranelli & Natali, 2005).

The correlation matrix is presented in Appendix B. The corresponding results, reported in Table 5, revealed overall moderate to very high correlations between the DAP-T tasks and their respective criterion measures, with raw Spearman’s ρ coefficients ranging from 0.43 (Golden Tokens Game—Counting Task, BVN) to 0.91 (Letter Game—Letter Naming Task, SPEED) which are indicative of moderate to large associations (Cohen, 2013). After correction for attenuation, the correlations ranged from 0.52 to 0.95, confirming the strength of the observed associations.

Tasks assessing alphabet knowledge, notational awareness, ANS and cardinality showed particularly high values, while measures non-symbolic numerical cognition abilities exhibited moderate-to-high correlations, confirming adequate agreement with established tools for the assessment of cognitive and pre-academic skills in preschool children. Overall, these findings provide strong support for the concurrent validity of the DAP-T tasks.

### 3.5. Analysis of Gender Differences

Before conducting the analyses, Levene’s test indicated that the assumption of homoscedasticity was met for all variables (*p* > .05; results shown in Appendix C). However, given that score distributions deviated from normality, gender differences were examined using the non-parametric Mann–Whitney U test. 

The results, reported in Table 6, indicated no statistically significant differences between males and females for most tasks. The only exception was the alphabet knowledge task (Letter Game), for which the Mann–Whitney U test revealed a significant difference (U = 816.500, *p* = .048), suggesting slightly better performance in male subjects (mean difference = −4.00). For all other tasks—Rhyme, RAN, Notational Awareness, Golden Tokens, ANS, and Cardinal Skills—the observed differences did not reach statistical significance (*p* > .05). Levene’s test confirmed homogeneity of variances between groups for all variables considered (*p* > .05), indicating that any differences found cannot be attributed to heterogeneity in score dispersion. 

### 3.6. Study of the Effect of Age

To examine the effect of age on performance across the DAP-T tasks, linear regression analyses were conducted using age in months (38–72 months) as a continuous predictor. The results, reported in Table 7, showed that age significantly predicted performance in all tasks. Standardized coefficients (β) ranged from 0.540 to 0.899 (all *p*-values < .001), indicating moderate to large age-related increases across the assessed skills. Explained variance was substantial for most tasks (R^2^ = 0.292–0.808), with the largest effects observed for Cardinality (β = 0.899, R^2^ = 0.808), Drawing and Writing (β = 0.834, R^2^ = 0.695), Letter Game (β = 0.790, R^2^ = 0.624), and Pathways (β = 0.785, R^2^ = 0.616). Moderate effects were found for Rhyme Game (β = 0.658, R^2^ = 0.433), ANS (β = 0.647, R^2^ = 0.418), Golden Tokens (β = 0.545, R^2^ = 0.297), and RAN (β = 0.540, R^2^ = 0.292).

Overall, the regression analyses confirmed a linear and systematic increase in performance with age across all DAP-T tasks, supporting the developmental progression of the cognitive precursors assessed by the instrument.

## 4. Discussion

The aim of this study was to present preliminary data on the psychometric properties of the DAP-T, a touchscreen-based digital tool designed to assess cognitive domains involved in the development of school learning processes in preschool age. The eight tasks included in the battery measure both domain-general precursors (visuomotor integration abilities) and domain-specific skills: for literacy, letter knowledge, phonological awareness, notational awareness, and RAN; for numeracy, non-symbolic abilities for quantity comparison and recognition, and symbolic abilities for counting and cardinality.

The preliminary analysis of item characteristics showed, in the overall sample, a generally low-to-moderate difficulty level. This result can be partly explained by the heterogeneity of the sample, which was predominantly composed of children in the 61–72 months age group, who are plausibly more competent in the skills assessed. The age-group analysis of item difficulty indicated that the initial items were the easiest, with a progressive increase in difficulty over the course of the task; moreover, average difficulty was higher in younger children (38–48 months) and progressively decreased with age. Linear regression analyses confirmed this developmental pattern, showing significant age-related increases in performance across all tasks. These findings support the idea that, in preschool-age children, it is possible to observe a progressive development of cognitive abilities related to academic skills (Aunola et al., 2004). 

Regarding item discrimination, discrimination index (DI) values indicated, in most tasks and age groups, a good ability of items to distinguish between high- and low-performing subjects, with few cases requiring revision or elimination. An exception was found in the Drawing and Writing Game, where a ceiling effect was observed in the 61–72 months group: for these children, items were too easy, and the low DI (<0.19) did not allow adequate discrimination of performance. A possible modification to this task could involve a more articulated scoring system, capable of rewarding the production of fully correct words and capturing more advanced orthographic skills (e.g., adherence to complex phonological rules), in line with developmental models of writing (e.g., Ferreiro & Teberosky, 1982). In future studies, it would also be advisable to consider sociodemographic variables such as family composition and parental education level, which may influence school readiness (Davis-Kean, 2005; Carneiro et al., 2013). 

The factor structure analysis using SEM confirmed that the DAP-T tasks can be represented by a single latent factor, supporting the presence of a general underlying ability. However, a two-factor model distinguishing literacy from numeracy tasks also yielded good fit indices. While the unifactorial solution was retained for parsimony in the present study, future research with larger samples should further investigate the possibility of a multidimensional structure. Internal consistency indices (ω = 0.713–0.966) indicated good reliability across all tasks. Concurrent validity analyses, corrected for attenuation of Spearman’s coefficients, revealed associations ranging from moderate to large (Cohen, 2013) with the criterion measures, confirming that the DAP-T assesses constructs consistent with those measured by established gold-standard tools in clinical practice.

A key finding of this study concerns the innovative potential of using touchscreen-based digital tools in developmental assessment. From an empirical perspective, the digital format was found to promote strong compliance among children, thanks to the interactive and game-like nature of the tasks (Papadakis et al., 2018; Neumann & Neumann, 2017). Furthermore, the use of digitized modalities allowed for the immediate processing of data, resulting in a substantial saving of time and resources compared to traditional paper-based procedures.

From an applied standpoint, the preliminary results obtained with the DAP-T highlight the potential of establishing a fast, standardized, and effective tool for preschool assessment. The ability to identify possible difficulties in the precursors of academic learning at an early stage enables the planning of targeted and timely educational and clinical interventions, thereby increasing the likelihood of positive developmental outcomes and reducing the risk of school failure.

The use of the DAP-T can represent a valuable resource for clinicians, teachers, and school professionals, providing relevant information to support the child’s development in a personalized way and to address school-related challenges more effectively. In this perspective, the tool can serve as a bridge between assessment and intervention, facilitating the design and implementation of educational and rehabilitative programs based on objective, immediately available data.

This study has several limitations. First, the small sample size, combined with the unbalanced distribution across age groups, limits the generalizability of the findings and may have affected the outcomes of statistical analyses, particularly those involving SEM techniques. In particular, the predominance of children in the 61–72 months group may have contributed to the generally low-to-moderate difficulty levels observed in several tasks. Moreover, because all tasks were administered on the same touchscreen device and within a single, homogeneous school setting, a potential common-method variance cannot be ruled out. Although this standardized administration ensured consistency, shared technological and contextual features may have inflated associations among tasks to some extent.

Secondly, the cross-sectional design and the absence of test–retest measures prevent the evaluation of predictive validity and long-term reliability. Furthermore, participants were recruited from a single geographical area, which restricts the applicability of the results to other contexts. Finally, the exclusive use of accuracy indices as performance measures may represent an additional limitation, especially considering that tablet-based administration allows for the recording and analysis of temporal parameters, which could provide valuable insights. 

Future studies should address these limitations by balancing the sample composition, including temporal and longitudinal measures, and examining the stability of the measurements over time.

## 5. Conclusions

The present study provided preliminary evidence supporting the psychometric properties of the DAP-T, a touchscreen-based tool for assessing both domain-general and domain-specific precursors of academic learning in preschool children. The analyses indicated appropriate item discrimination, adequate internal reliability, and strong evidence of concurrent validity with established criterion measures. The unidimensional factor structure further support the use of the DAP-T as a reliable tool for evaluating early cognitive abilities.

Despite limitations related to sample size and composition, the findings suggest that the DAP-T represents a promising option for early screening and research in educational and clinical contexts. Further longitudinal studies with larger samples and longitudinal designs will be needed to confirm predictive validity and the generalizability of the results.

## Figures and Tables

**Table 1:** Demographic characteristics of the sample.

	N	%	Mean Age (SD)	IQR
Total N	105	100	61.43 (10.38)	54–69
Females	56	53.3	61.14 (10.43)	54–69
Males	49	46.7	61.75 (10.42)	54–69
36–48 months	18	17.1		
49–60 months	20	19.0		
61–72 months	67	63.8		

**Table 2:** Descriptives of the sample.

	Letter Game	Rhyme Game	Rapid Automatized Naming (RAN) of Pictures	Drawing and Writing Game	Golden Tokens Game	Quantity Comparison (ANS)	Ball Jar Game	Pathways Game
N	103	100	93	102	96	101	94	105
Missing	2	5	12	3	9	4	11	0
Mean	21.060	9.670	45.871	20.480	3.125	8.594	37.245	88.267
Median	27.000	9.500	46	25.000	3.000	9	58.000	95
Standard deviation	12.498	3.766	15.515	8.814	2.048	1.698	23.930	16.625
Minimum	0.000	1.000	1	0	0	3	0	2
Maximum	35.000	16.000	78	27	6	10	58	104
Skewness	−0.584	−0.082	−0.282	−1.248	−0.060	−1.490	−0.449	−2.088
Std. error skewness	0.238	0.241	0.250	0.239	0.246	0.240	0.249	0.236
Kurtosis	−1.234	−0.983	−0.049	0.033	−1.360	1.730	−1.605	6.353
Std. error kurtosis	0.472	0.478	0.495	0.474	0.488	0.476	0.493	0.467
Shapiro–Wilk W	0.846	0.962	0.990	0.733	0.904	0.786	0.746	0.789
Shapiro–Wilk p	<0.001	0.006	0.688	<0.001	<0.001	<0.001	<0.001	<0.001

**Table 3:** SEM Model Results.

Variables	Standardized Factor Loadings (CI: 95%)	Standard Errors (SEs)	Residuals
Letter Game (letter knowledge)	0.802 * (0.695–0.908)	0.054	0.357
Rhyme Game (phonological awareness)	0.723 * (0.622–0.825)	0.052	0.477
Rapid Automatized Naming (RAN)	0.737 * (0.618–0.856)	0.061	0.457
Drawing and Writing Game (Notational Awareness)	0.814 * (0.722–0.906)	0.047	0.337
Quantity Comparison (ANS)	0.578 * (0.414–0.742)	0.084	0.666
Golden Tokens Game (Quantity Recognition)	0.607 * (0.465–0.748)	0.072	0.632
Ball Jar Game (Cardinal Principle)	0.754 * (0.649–0.859)	0.054	0.431
Pathways Game (Visuomotor Integration)	0.657 * (0.537–0.778)	0.061	0.568

Note. * p < .001. CI = Confidence Intervals.

**Table 4:** Reliability McDonald’s ω coefficient of DAP-T tasks.

Task	ω Value
Letter Game *(letter knowledge)*	0.964
Rhyme Game *(phonological awareness)*	0.808
Rapid Automatized Naming *(RAN)*	0.964
Drawing and Writing Game *(Notational Awareness)*	0.953
Quantity Comparison *(ANS)*	0.713
Golden Tokens Game *(Quantity Recognition)*	0.772
Ball Jar Game *(Cardinal Principle)*	0.966
Pathways Game *(Visuomotor Integration)*	0.814

**Table 5:** Correlation indices between DAP-T task and Criterion measures.

Construct	DAP-T Task	Criterion Measure	Correlation Coefficient (*n*)	Correlation Corrected for Attenuation
Letter Knowledge	Letter Game	Letter Recognition (SPEED)	ρ = 0.84 *** (*n* = 58)	0.95
		Letter Naming (SPEED)	ρ = 0.91 *** (*n* = 55)	0.95
Phonological Awareness	Rhyme Game	Rhyme Recognition (CMF)	ρ = 0.64 *** (*n* = 53)	0.85
Rapid Naming	Rapid Automatized Naming (RAN) of Pictures	Rapid Naming (RIAS-2)	ρ = 0.55 *** (*n* = 44)	0.58
Notational Awareness	Drawing and Writing Game	Letter Writing (SPEED)	ρ = 0.76 *** (*n* = 54)	0.81
ANS	Quantity Comparison	Quantity Comparison (BIN 4-6)	ρ = 0.60 *** (*n* = 57)	0.86
Quantity Recognition	Golden Tokens Game	Quantity Comparison (BIN 4-6)	ρ = 0.56 *** (*n* = 53)	0.89
		Counting Graphic Elements (BVN 5-11)	ρ = 0.43 ** (*n* = 51)	0.52
Cardinal Principle	Ball Jar Game	Quantity Comparison (BIN 4-6)	ρ = 0.72 *** (*n* = 53)	0.86
		Counting Graphic Elements (BVN 5-11)	ρ = 0.61 *** (*n* = 51)	0.65
Visuomotor Integration	Pathways Game	VMI	ρ = 0.55 *** (*n* = 61)	0.62

Notes. ** p < .01, *** p < .001.

**Table 6:** Mann–Whitney U test for gender differences study.

Independent Samples *t*-Test		Statistics	*p*	Mean Difference	Effect Size
Letter Game	Mann–Whitney U test	816.500	0.048 *	−4.000	0.238
Rhyme Game	Mann–Whitney U test	1186.000	0.803	−0.000	0.029
Rapid Automatized Naming (RAN) of Pictures	Mann–Whitney U test	975.500	0.360	−3.000	0.110
Drawing and Writing Game	Mann–Whitney U test	1044.000	0.529	−0.000	0.074
Golden Tokens Game	Mann–Whitney U test	1097.000	0.818	0.000	−0.015
Quantity Comparison (ANS)	Mann–Whitney U test	1060.000	0.299	−0.000	0.117
Ball Jar Game	Mann–Whitney U test	738.500	0.498	0.000	−0.087
Pathways Game	Mann–Whitney U test	1151.500	0.772	−0.000	0.037

*Note. Alternative hypothesis (Hₐ): μF ≠ μM. An asterisk indicates a statistically significant result (p < .05). Note 2. Effect size is reported as rank-biserial correlation (r).*

**Table 7:** Linear regression analyses examining the effect of age on DAP-T task performance.

Tasks	N	R^2^	B (SE)	β [95% CI]	*p*
Letter Game	93	0.624	0.962 (0.078)	0.790 [0.663, 0.918]	<.001
Rhyme Game	99	0.433	0.244 (0.028)	0.658 [0.507, 0.810]	<.001
Rapid Automatized Naming (RAN) of Pictures	94	0.292	0.905 (0.147)	0.540 [0.366, 0.714]	<.001
Drawing and Writing Game	95	0.695	0.694 (0.048)	0.834 [0.720, 0.947]	<.001
Golden Tokens Game	94	0.297	0.113 (0.018)	0.545 [0.371, 0.719]	<.001
Quantity Comparison (ANS)	98	0.418	0.103 (0.012)	0.647 [0.492, 0.801]	<.001
Ball Jar Game	79	0.808	1.967 (0.109)	0.899 [0.800, 0.998]	<.001
Pathways Game	97	0.616	1.041 (0.084)	0.785 [0.658, 0.911]	<.001

## Data Availability

The data presented in this study are available on request from the corresponding author.

## Abbreviations

The following abbreviations are used in this manuscript:

DAP-T	Developmental Assessment for Preschoolers—Tool
RAN	Rapid Automatized Naming
ANS	Approximate Number System

## Appendix A. Item Difficulty Analysis and Discrimination Index (DI)

**Table:**

**Letter Game**				**Proportion Correct (Item Difficulty)**													
**Sample/Subsample**	**N**	**Item 1**	**Item 2**	**Item 3**	**Item 4**	**Item 5**	**Item 6**	**Item 7**	**Item 8**	**Item 9**	**Item 10**	**Item 11**	**Item 12**	**Item 13**	**Item 14**	**Item 15**	**Item 16**
**General (38–72 months)**	103	87.38	66.99	71.84	68.93	66.02	76.70	64.08	67.96	58.25	67.96	69.90	71.84	66.99	62.14	62.14	59.22
**36–48 months**	17	29.41	29.41	23.53	17.65	23.53	29.41	17.65	35.29	5.88	23.53	29.41	17.65	11.76	17.65	29.41	5.88
**49–60 months**	20	100.00	60.00	60.00	50.00	50.00	65.00	50.00	55.00	55.00	60.00	55.00	65.00	55.00	55.00	45.00	30.00
**61–72 months**	66	98.48	78.79	87.88	87.88	81.82	92.42	80.30	80.30	72.73	81.82	84.85	87.88	84.85	75.76	75.76	81.82
		**Discrimination Index**															
**General**		0.48	0.93	0.85	0.96	0.89	0.81	1.00	0.93	0.93	0.93	0.96	0.89	0.81	0.96	0.85	0.96
**36–48 months**		1.00	1.00	0.75	0.75	0.75	0.75	0.75	1.00	0.25	1.00	1.00	0.75	0.50	0.75	1.00	0.25
**49–60 months**		0.00 *	1.00	1.00	1.00	1.00	1.00	1.00	1.00	1.00	1.00	1.00	0.80	0.80	1.00	0.80	0.80
**61–72 months**		0.06 *	0.76	0.35	0.35	0.65	0.24	0.59	0.71	0.82	0.65	0.59	0.47	0.41	0.76	0.82	0.65
**Rhyme Game**				**Proportion Correct (Item Difficulty)**													
**Sample/Subsample**	**N**	**Item 1**	**Item 2**	**Item 3**	**Item 4**	**Item 5**	**Item 6**	**Item 7**	**Item 8**	**Item 9**	**Item 10**	**Item 11**	**Item 12**	**Item 13**	**Item 14**	**Item 15**	**Item 16**
**General (38–72 months)**	100	80.20	70.30	86.14	68.32	81.19	83.17	60.40	57.43	49.00	48.51	53.47	61.39	39.60	50.50	45.54	34.65
**36–48 months**	16	56.25	43.75	81.25	50	62.5	68.75	12.5	25	18.75	12.5	18.75	18.75	12.5	18.75	31.25	18.75
**49–60 months**	19	84.21	52.63	73.68	57.89	57.89	68.42	21.05	57.89	42.11	31.58	31.58	47.37	47.37	36.84	42.11	31.58
**61–72 months**	65	84.85	81.82	90.91	75.76	92.42	90.91	83.33	65.15	57.58	62.12	68.18	75.76	43.94	62.12	50.00	39.39
		**Discrimination Index**															
**General**		0.52	0.56	0.26	0.48	0.33	0.33	0.85	0.78	0.63	0.81	0.96	0.63	0.63	0.74	0.52	0.48
**36–48 months**		0.75	0.25	0.50	0.25	0.00	−0.25 *	−0.25 *	0.50	0.00	0.00	0.50	0.50	0.25	0.25	0.50	0.75
**49–60 months**		0.40	0.80	0.60	0.20	0.40	0.60	0.40	0.60	0.60	0.60	0.60	0.40	0.80	0.60	0.60	0.00 *
**61–72 months**		0.47	0.35	0.24	0.35	0.24	0.24	0.47	0.82	0.65	0.47	0.76	0.59	0.41	0.47	0.59	0.41
**Drawing and Writing Game**				**Proportion Correct (Item Difficulty)**													
**Sample/Subsample**	**N**	**Item 1**	**Item 2**	**Item 3**	**Item 4**	**Item 5**	**Item 6**	**Item 7**	**Item 8**	**Item 9**	**Item 10**	**Item 11**	**Item 12**	**Item 13**	**Item 14**	**Item 15**	
**General (38–72 months)**	102	93.88	97.96	95.92	85.71	85.71	84.69	82.65	84.69	82.65	82.65	81.63	81.63	81.63	78.57	79.59	
**36–48 months**	18	75	87.5	81.25	25	31.25	25	18.75	37.5	31.25	31.25	25	31.25	25	25	25	
**49–60 months**	20	95	100	95	95	90	85	80	80	75	70	75	65	70	60	65	
**61–72 months**	64	98.39	100.00	100.00	98.39	98.39	100.00	100.00	98.39	98.39	100.00	98.39	100.00	100.00	98.39	98.39	
		**Discrimination Index**															
**General**		0.15 *	0.08 *	0.12 *	0.50	0.50	0.58	0.65	0.54	0.62	0.65	0.65	0.65	0.69	0.69	0.69	
**36–48 months**		0.25	0.25	0.00	0.75	0.50	0.50	0.75	0.75	0.75	1.00	0.75	1.00	0.75	0.75	0.75	
**49–60 months**		0.00 *	0.00 *	0.00 *	0.20	0.40	0.60	0.80	0.80	1.00	1.00	0.80	1.00	1.00	1.00	1.00	
**61–72 months**		0.06 *	0.00 *	0.00 *	0.06 *	0.06 *	0.00 *	0.00 *	0.06 *	0.06 *	0.00 *	0.00 *	0.00 *	0.00 *	0.06 *	0.06 *	
**Quantity Comparison (ANS) Task**		**Proportion Correct (Item Difficulty)**															
**Sample/Subsample**	**N**	**Item 1**	**Item 2**	**Item 3**	**Item 4**	**Item 5**	**Item 6**	**Item 7**	**Item 8**	**Item 9**	**Item 10**						
**General (38–72 months)**	101	92.08	94.06	96.04	95.05	69.31	86.14	87.13	87.13	84.16	69.31						
**36–48 months**	16	56.25	81.25	75	81.25	68.75	75	75	68.75	62.5	50						
**49–60 months**	19	94.44	94.44	100.00	88.89	55.56	77.78	72.22	72.22	72.22	55.56						
**61–72 months**	66	100.00	97.01	100.00	100.00	73.13	91.04	94.03	95.52	92.54	77.61						
		**Discrimination Index**															
**General**		0.26	0.22	0.11 *	0.15 *	0.52	0.37	0.41	0.48	0.48	0.67						
**36–48 months**		0.50	0.50	0.50	0.25	0.25	0.75	0.25	0.75	0.25	0.75						
**49–60 months**		0.25	0.00 *	0.00 *	0.50	0.25	0.75	0.50	0.75	0.75	0.25						
**61–72 months**		0.00 *	0.11 *	0.00 *	0.00 *	0.61	0.22	0.22	0.17 *	0.28	0.56						
**Golden Tokens Game**		**Proportion Correct (Item Difficulty)**															
**Sample/Subsample**	**N**	**Item 1**	**Item 2**	**Item 3**	**Item 4**	**Item 5**	**Item 6**										
**General (38–72 months)**	96	75.00	61.96	51.09	45.65	47.83	43.48										
**36–48 months**	15	63.64	45.45	9.09	9.09	18.18	9.09										
**49–60 months**	18	64.71	58.82	35.29	23.53	29.41	29.41										
**61–72 months**	63	79.69	65.63	62.50	57.81	57.81	53.13										
		**Discrimination Index**															
**General**		0.71	0.79	0.88	0.71	0.96	0.96										
**36–48 months**		1.00	0.67	0.33	0.33	0.33	0.33										
**49–60 months**		1.00	0.50	0.50	0.50	0.75	0.50										
**61–72 months**		0.53	0.94	0.94	0.76	0.82	0.88										
**Ball Jar Game**		**Proportion Correct (Item Difficulty)**															
**Sample/Subsample**	**N**	**Item 1**	**Item 2**	**Item 3**	**Item 4**	**Item 5**	**Item 6**	**Item 7**	**Item 8**	**Item 9**	**Item 10**	**Item 11**	**Item 12**				
**General (38–72 months)**	94	96.77	89.25	88.17	81.72	75.27	75.27	66.67	63.44	63.44	59.14	55.91	54.84				
**36–48 months**	16	84.62	46.15	46.15	38.46	15.38	15.38	7.69	0.00	0.00	0.00	0.00	0.00				
**49–60 months**	16	100.00	93.33	93.33	73.33	66.67	66.67	40.00	40.00	40.00	33.33	20.00	20.00				
**61–72 months**	62	100.00	98.36	96.72	96.72	95.08	95.08	90.16	86.89	86.89	81.97	80.33	78.69				
		**Discrimination Index**															
**General**		0.12 *	0.4	0.44	0.68	0.92	0.92	1	1	1	1	1	1				
**36–48 months**		0.67	1.00	1.00	1.00	0.67	0.67	0.33	0.00 *	0.00 *	0.00 *	0.00 *	0.00 *				
**49–60 months**		0.00 *	0.25	0.25	1.00	1.00	1.00	1.00	1.00	1.00	1.00	0.75	0.75				
**61–72 months**		0.00 *	0.06 *	0.13 *	0.13 *	0.19	0.19	0.38	0.50	0.50	0.69	0.75	0.81				

Note. Values marked with * indicate DI < 0.19.

## Appendix B. Matrix of Correlations Between DAP-T and Criterion Measures

**Table:**

		1	2	3	4	5	6	7	8	9	10	11	12	13	14	15
**1. DAP-T LG**	**ρ**	—														
	**df**	—														
**2. DAP-T RG**	**ρ**	0.605 ***	—													
	**df**	97	—													
**3. DAP-T RAN**	**ρ**	0.612 ***	0.472 ***	—												
	**df**	90	88	—												
**4. DAP-T DWG**	**ρ**	0.756 ***	0.684 ***	0.545 ***	—											
	**df**	99	96	89	—											
**5. DAP-T ANS**	**ρ**	0.383 ***	0.386 ***	0.389 ***	0.408 ***	—										
	**df**	98	96	89	97	—										
**6. DAP-T GTG**	**ρ**	0.456 ***	0.430 ***	0.460 ***	0.530 ***	0.389 ***	—									
	**df**	93	92	85	92	94	—									
**7. DAP-T BJG**	**ρ**	0.581 ***	0.475 ***	0.363 ***	0.569 ***	0.477 ***	0.476 ***	—								
	**df**	91	90	83	91	92	88	—								
**8. DAP-T PWG**	**ρ**	0.477 ***	0.402 ***	0.293 **	0.415 ***	0.393 ***	0.345 ***	0.529 ***	—							
	**df**	101	98	91	100	99	94	92	—							
**9. SPEED-1**	**ρ**	0.842 ***	0.418 **	0.563 ***	0.646 ***	0.430 ***	0.525 ***	0.671 ***	0.500 ***	—						
	**df**	56	55	47	57	54	53	51	57	—						
**10. SPEED-2**	**ρ**	0.908 ***	0.344 *	0.468 **	0.593 ***	0.365 **	0.368 **	0.649 ***	0.473 ***	0.880 ***	—					
	**df**	53	51	44	53	50	49	48	53	53	—					
**11. SPEED-3**	**ρ**	0.860 ***	0.516 ***	0.650 ***	0.762 ***	0.528 ***	0.597 ***	0.686 ***	0.633 ***	0.881 ***	0.848 ***	—				
	**df**	52	50	43	52	49	48	47	52	52	52	—				
**12. CMF**	**ρ**	0.468 ***	0.639 ***	0.533 ***	0.599 ***	0.483 ***	0.391 **	0.398 **	0.376 **	0.378 **	0.389 **	0.579 ***	—			
	**df**	50	51	43	51	50	49	48	51	51	49	49	—			
**13. RIAS-2**	**ρ**	0.519 ***	0.275	0.552 ***	0.423 **	0.572 ***	0.388 **	0.512 ***	0.384 **	0.541 ***	0.464 **	0.558 ***	0.275	—		
	**df**	45	45	42	45	44	44	42	45	44	44	43	43	—		
**14. BIN**	**ρ**	0.502 ***	0.569 ***	0.406 **	0.548 ***	0.604 ***	0.558 ***	0.720 ***	0.540 ***	0.545 ***	0.449 ***	0.581 ***	0.439 **	0.399 **	—	
	**df**	53	53	45	54	53	51	51	54	52	50	49	50	44	—	
**15. BVN**	**ρ**	0.599 ***	0.263	0.436 **	0.449 ***	0.470 ***	0.434 **	0.607 ***	0.420 **	0.620 ***	0.578 ***	0.651 ***	0.299 *	0.486 ***	0.571 ***	—
	**df**	51	50	43	51	50	49	48	51	50	49	48	48	44	51	—
**16. VMI**	**ρ**	0.560 ***	0.518 ***	0.510 ***	0.751 ***	0.549 ***	0.471 ***	0.688 ***	0.548 ***	0.638 ***	0.556 ***	0.666 ***	0.556 ***	0.573 ***	0.567 ***	0.543 ***
	**df**	58	57	49	59	56	54	53	59	57	53	52	51	45	54	51

Notes. ** p < .05, ** p < .01, *** p < .001; ρ = Spearman’s Rho; df = degrees of freedom.* DAP-T tasks: *DAP-T LG = Letter Game; DAP-T RG = Rhyme Game; DAP-T RAN = Rapid Automatized Naming of Pictures; DAP-T DWG = Drawing and Writing Game; DAP-T ANS = Quantity Comparison; DAP-T GTG = Golden Tokens Game; DAP-T BJG = Ball Jar Game; DAP-T PWG = Pathways Game.* Criterion measures: *SPEED-1 = Letter Recognition; SPEED-2 = Letter Naming; SPEED-3 = Letter Writing; CMF = Rhyme Recognition; RIAS-2 = Rapid Naming; BIN = Quantity Comparison; BVN = Counting Graphic Elements; VMI = Visual-Motor Integration.*

## Appendix C. Results of Levene’s Test for Homogeneity of Variances Across Gender for the DAP-T Tasks

**Table:**

Variable	F	df	df2	*p*
Letter Game	0.109	1	91	0.742
Rhyme Game	0.370	1	97	0.544
Rapid Automatized Naming (RAN) of Pictures	0.040	1	92	0.841
Drawing and Writing Game	0.509	1	93	0.477
Golden Tokens Game	0.658	1	93	0.419
Quantity Comparison (ANS)	0.267	1	96	0.606
Ball Jar Game	1.856	1	78	0.177
Pathways Game	0.047	1	96	0.828

*Note. A small p value suggests a violation of the equal variances assumption.*
